# Retrograde intrarenal surgery technique without using fluoroscopy and access sheet in the treatment of kidney stones

**DOI:** 10.3906/sag-1811-152

**Published:** 2019-06-18

**Authors:** Fatih FIRDOLAŞ, Necip PİRİNÇÇİ, Tunç OZAN, Ahmet KARAKEÇİ, İrfan ORHAN

**Affiliations:** 1 Department of Urology, Faculty of Medicine, Fırat University, Elazığ Turkey

**Keywords:** Kidney stone, fluoroscopy, retrograde intrarenal surgery

## Abstract

**Background/aim:**

In this study, we aimed to present our results on single-guidewire flexible ureteroscopy and retrograde intrarenal surgery without fluoroscopy and an access sheet, and to evaluate the efficacy and safety of this procedure retrospectively.

**Material and methods:**

Our routine technique can be described as the evaluation of the ureter using a semirigid ureterorenoscope (URS), leading in the guidewire through the semirigid URS, pulling the semirigid URS back, inserting the flexible URS with the aid of the guidewire, inserting the laser probe through the flexible URS, and performing laser lithotripsy.

**Results:**

Our study included 400 male and 198 female patients with a mean age of 36.8 ± 16 (14–80) years. The mean stone size was 8.7 ± 4 (8–20) mm, and the mean operation time was 56 (32–106) min. Postoperative fever was observed in 24 (4%) of the patients, and 30 (5%) patients had hematuria as a minor complication. A stone-free status was observed in 466 (78%) patients, while 102 (17%) patients had clinically insignificant minor stone fragments and 30 patients had clinically significant stone residue.

**Conclusion:**

The retrograde intrarenal surgery procedure using only a guidewire without fluoroscopy and an access sheet in the treatment of kidney stones is technically safe and effective.

## 1. Introduction

The primary aim in the treatment of renal stones is to achieve minimum morbidity and maximum stone-free status. Treatment modalities for kidney stones have changed significantly in recent years, and alternative, minimally invasive methods have gained importance. While in past years kidney stone treatment has been performed only through open surgery, less invasive surgical methods are now frequently used, including percutaneous nephrolithotomy, extracorporeal shock wave lithotripsy (ESWL), and retrograde intrarenal surgery (RIRS) (1). 

The development of small ureterorenoscope (URS) devices and improvements in their deflection angles and optical systems have made the RIRS procedure popular in the treatment of kidney stones (2). In the classical application of RIRS, fluoroscopy is needed for the insertion of the access sheet, allowing for the placement of the guidewire and an easy approach to the kidney stone (3,4). Because of exposure to radiation during fluoroscopy, both the patient and the surgeon are at risk of developing pathologies such as secondary tumors, infertility, and genetic mutations (5,6). The use of a ureteral access sheet can increase the duration of fluoroscopy and therefore the exposure to radiation, and may cause ureteral injury. For this reason, there has been a recent search for different techniques to reduce exposure to radiation during RIRS as well as other complications. In this study, we aimed to evaluate the results and benefits of using the RIRS technique without fluoroscopy or an access sheet. 

## 2. Materials and methods 

Ethics approval for the study was obtained from the local ethics committee. A total of 598 patients (400 males and 198 females) were evaluated retrospectively after treatment for kidney stones using the RIRS procedure without fluoroscopy and an access sheet between March 2014 and June 2018. The diagnoses of urolithiasis were based on preoperative imaging methods (plain radiography, ultrasonography [USG], and low-dose computed tomography [CT]). Stone size was determined based on surface area, which was calculated according to the European Association of Urology guidelines. The treatment method was applied to patients with ESWL-refractory stones smaller than 2 cm in diameter. Patients with a bifid pelvis, ectopic pelvic fusion abnormalities, or calyceal diverticulum stones were excluded from the study. The demographic data of the patients, the sizes and sites of the stones, the operation durations, the stone-free status rates, and the hospital stay durations were recorded. A surgical consent form was obtained from all patients before surgery. The complete blood counts, serum creatinine levels, bleeding and clotting times, urinalyses, and urine cultures of the patients were recorded. A single preoperative dose of 1 g of ceftriaxone was routinely administered. The patients with positive urine cultures were given appropriate antibiotic treatment before surgery and negative cultures were obtained for all patients before surgery. The main outcomes assessed were stone-free status and complication rates at the first month after a single procedure. All patients underwent kidney, ureter, and bladder radiography on the first day and low-dose CT at the first postoperative month. The results were classified as “stone-free,” “clinically insignificant residual fragments (CIRFs),” or “residual stones.” Stone-free status was defined as the absence of any fragments. CIRFs were defined as nonobstructing, noninfectious, and asymptomatic residual fragments of ≤4 mm (7). Residual stones were defined as stones with a diameter of >4 mm or stones with symptomatic features (7).

### 2.1. Surgical technique

All surgical procedures were applied under general anesthesia in the lithotomy position. The evaluation of the ureter and any additional ureter stones and the placement of the guidewire before RIRS were performed with a semirigid ureterorenoscope (Wolf, Germany). The guidewire (0.9652 mm, hydrophilic material, coated, flexible-type guidewire, Cook Medical, Limerick, Ireland) was placed into the renal pelvis with the ureterorenoscope. The guidewire was then inserted into the working channel of the 7.5-F flexible ureterorenoscope (Storz Flex-X2, Tuttlingen, Germany), and the flexible ureterorenoscope was pushed forward, using the tension of the guidewire, into the ureter until the renal pelvis came under visualization. In cases of ureteral orifice stricture, where we were unable to proceed into the ureter, the orifice was dilated with balloon dilatation. In cases where the ureterorenoscope was unable to proceed despite dilatation, a double-J stent was placed and the procedure was repeated after 1 month. After the renal pelvis had come under visualization, the guidewire was removed, and a 272-µm laser fiber of a holmium laser device (Wolf, Germany) was inserted. The energy level of 1.0–2.0 J and a rate of 5–10 Hz was used for stone dusting in all patients. At the end of the procedure, the flexible ureterorenoscope was pulled out under visualization while the ureter was observed so that no possible injury was missed. For patients with solitary kidneys, mucosal edema, injury, or a heavy stone burden, a 4.8-F, 28-cm double-J stent was inserted through the semirigid ureterorenoscope. Complications were scored according to the modified Clavien–Dindo classification (8). According to this classification, complications of the first degree are described as complications delaying discharge of the patient without requiring any additional intervention. Second degree complications are complications needing medical treatment. Third degree complications require surgical, endoscopic or radiological intervention. Fourth degree complications are described as life-threatening complications, and exitus of the patient is termed as complication of the fifth grade (8).

### 2.2. Statistical analysis

The demographic and operational data of the patients are presented as means ± standard deviations. The statistical analysis was performed with SPSS 18.0 for Windows (Chicago, IL, USA). For comparisons of the categorical variables, the chi-square test was used, and for comparisons of the 2 groups, Student’s t-test was used. The confidence interval was set at 95%, and P < 0.05 was considered statistically significant.

## 3. Results

The study included 400 (67%) male and 198 (33%) female patients with a mean age of 36.8 ± 16 (14–80) years. The surgeries were performed after negative preoperative urine cultures were obtained for all patients. The mean stone diameter was 8.7 ± 4 (8–20) mm, and the mean operation time was 56 ± 15(32–106) min. The flexible URS could not proceed through the ureter orifice in 30 patients, and a double-J stent was therefore placed and the surgery was repeated 1 month later. The stone characteristics are presented in Table 1. No intraoperative complications were observed in any of the patients. Postoperative fever was observed in 24 (4%) patients (Modified Clavien 1), and 30 (5%) of the patients showed hematuria as a minor complication (Modified Clavien 2). The perioperative outcomes are described in Table 2. Stone-free status was observed in 466 (78%) patients, while 102 (17%) patients had clinically insignificant minor stone fragments and 30 (5%) patients had clinically significant stone residue. The success rates according to stone location are provided in Table 3. For all patients with no clinically significant residual stone fragments (n = 568, 95%), the double-J stent was removed under local anesthesia, and for the patients with clinically significant residual stone fragments (n = 30, 5%), the double-J stent was removed under general anesthesia by the performance of a diagnostic URS procedure. 

**Table 1:** Demographic and clinical data.

Variable	No. of cases (%)	Mean ± SD (range)
Operative time (min)		56 (32–106)
Stone-free rate	466 (78)	
CIRF	102 (17)	
Residual stone	30 (5)	
Complication Fever Hematuria	24 (4) 30 (5)	

**Table 2:** Perioperative outcomes.

Variable	No. of cases (%)	Mean ± SD (range)
Age, years		36.8 ± 16 (14–80)
Sex Male Female	400 (67) 198 (33)	
Laterality Left Right	287 (48) 311 (52)	
Stone size, mm		8.7 ± 4 (8–20)
Stone locationPelvis Lower calyx Middle calyxUpper calyx Ureter	341 (57) 185 (31) 42 (7) 30 (5)18 (3)	

CIRF: Clinically insignificant residual fragments; SD: standard deviation.

**Table 3:** Success rates according to stone location.

	Stone-free (%)	CIRF (%)	Residual stone (%)	Total (%)
Renal pelvis	286 (84)	41 (12)	14 (4)	341 (57)
Lower pole	115 (62)	56 (30)	14 (8)	185 (31)
Middle pole	38 (91)	3 (7)	1 (2)	42 (7)
Upper pole	27 (90)	2 (7)	1 (3)	30 (5)
Total (%)	466 (78)	102 (17)	30 (5)	598 (100)

CIRF: Clinically insignificant residual fragments.

## 4. Discussion

In a multicenter study performed in Turkey, the prevalence and incidence of stone disease were reported as 14.8% and 2.2%, respectively (9). It was reported that the disease is encountered frequently in patients aged in their 30s and 40s, and it is 1.5 times more common in males and in people with low socioeconomic status and low education. It is reported that there is no difference between inhabitants of rural and metropolitan areas (9). The RIRS procedure is one of the current treatment modalities for renal stone disease. The first RIRS procedure, performed in 1983 by Huffman et al. for the treatment of renal pelvis stones, used a rigid URS and ultrasonic lithotripter (10). In 1995, the introduction of the holmium laser in RIRS was considered a milestone in renal stone treatment. Thanks to this evolution, all types of renal stones were treated with success regardless of their composition (11). The major advantages of this procedure are 100% stone fragmentation and disposal and a short operation time (12). The biggest disadvantage of the RIRS procedure is that it is performed under fluoroscopy. A ureteral access sheet is frequently used to extend the lifetime of the URS device and facilitate multiple entries during one session; however, injuries related to access sheet use are another possible disadvantage. Radiation exposure during access sheet placement and the RIRS procedure increases because of the increased duration of the fluoroscopy (13).

The use of fluoroscopy during RIRS plays a key role in promoting the safety of the procedure (14). However, despite its advantages, fluoroscopy may cause pathologies such as cancer, infertility, and genetic mutations in the patients or the surgical team (13). The severity of these potential effects is related to the dosage and duration of the radiation exposure; therefore, the use of protective equipment is critical in minimizing these effects. However, despite all precautions, exposure to the harmful effects of radiation during RIRS is inevitable, especially for patients but also for the surgery team (15). 

The first step in the classic RIRS procedure is to place the guidewire safely. We performed this step under direct visualization with the semirigid URS until the renal pelvis was visualized. Placing the ureteral access sheet is classically done by sliding it over the guidewire catheter under fluoroscopy. In the method we applied, we did not use a ureteral access sheet, dispensing with the need for fluoroscopy and thereby avoiding exposure to the harmful effects of radiation. 

The first study in the literature on RIRS without fluoroscopy, which consisted of a total of 110 patients with a mean age of 33.5 years (range: 12–65) and mean stone size of 8.7 mm (range: 6–15), included patients undergoing endoscopic treatment for distal ureter stones (16). In this study, fluoroscopy was only needed in 24 (4%) cases, and no complications were reported. Another study reported that fluoroscopy was needed in only 7.52% of cases. In this study, the mean age of patients was reported as 34.03 ± 12.09 years (range: 9–63) and the mean stone size was 10.64 ± 3.16 mm (range: 6–17). According to that study, patients underwent endoscopic treatment for mid- and proximal ureter stones and only minor complications were observed in 11% of all cases (17). In another study, where RIRS was applied to patients including 62 (66.6%) males and 31 (33.3%) females with a mean age of 47.8 ± 14 years (range: 14–93), mean stone size was reported as 14.7 ± 5 mm (range: 7–32). In this study, an ureteral access sheet was used, and it was reported that fluoroscopy was not used in any of the cases and that no serious complications were encountered (18). In another study by Kirac et al., it was reported that a single dose was used to decrease the use of fluoroscopy when verifying the location of the ureteral access sheet (19). The advantage of our study is that we proceeded to the renal pelvis under direct visualization and there was no need for the use of fluoroscopy. 

The use of an access sheet may have unfavorable results, such as ureteral injury and increased surgery costs (20). Traxer et al. reported mild ureteral injury in 33% of cases and serious ureteral injury in 13% of cases in their RIRS series, which consisted of 359 patients (21). In contrast, the advantage of our technique is that we proceeded until the renal pelvis came under direct visualization and prevented any complications due to the blind insertion of the ureteral access sheet. 

RIRS is accepted by many practicing authors as an effective and practicable method for the treatment of kidney stones (22). In their RIRS study, in which they used an access sheet without fluoroscopy, Peng et al. reported a stone-free rate of 95.7%, describing stone-free status as no visual stone fragments or stone fragments of <2 mm in kidney-ureter-bladder (KUB)-graphy or USG (23). In some studies, stone fragments have been removed with basket catheters or similar devices, while in our study, we chose the spontaneous passage of the stone fragments. Like many other studies, we also used KUB-graphy and USG for determining residual stone fragments (24–26). The stone-free status rate in our study, in which, unlike Peng at al., we did not use an access sheet, was 78%. 

Our study’s limitation is that we used a retrospective and incomparable method. However, this study shows that RIRS without fluoroscopy or an access sheet is effective, safe, and applicable. Future studies are needed to compare the standard technique to the technique without fluoroscopy. 

In conclusion, the RIRS procedure applied only on a guidewire without fluoroscopy and an access sheet is a safe, effective, and technically applicable method for the treatment of kidney stones and prevents exposure to radiation for the patient and the surgery team.
